# Construction of an endoplasmic reticulum stress-related gene model for predicting prognosis and immune features in kidney renal clear cell carcinoma

**DOI:** 10.3389/fmolb.2022.928006

**Published:** 2022-09-02

**Authors:** Yuanhao Shen, Yinghao Cao, Lei Zhou, Jianfeng Wu, Min Mao

**Affiliations:** ^1^ Department of Urology, Shanghai General Hospital, Shanghai Jiao Tong University School of Medicine, Shanghai, China; ^2^ Department of Orthopedics, Shanghai General Hospital, Shanghai Jiao Tong University School of Medicine, Shanghai, China

**Keywords:** kidney renal clear cell carcinoma, endoplasmic reticulum stress, prognosis, tumor microenvironment, immunotherapy

## Abstract

**Background:** Kidney renal clear cell carcinoma (KIRC) is one of the most lethal malignant tumors with a propensity for poor prognosis and difficult treatment. Endoplasmic reticulum (ER) stress served as a pivotal role in the progression of the tumor. However, the implications of ER stress on the clinical outcome and immune features of KIRC patients still need elucidation.

**Methods:** We identified differentially expressed ER stress-related genes between KIRC specimens and normal specimens with TCGA dataset. Then, we explored the biological function and genetic mutation of ER stress-related differentially expressed genes (DEGs) by multiple bioinformatics analysis. Subsequently, LASSO analysis and univariate Cox regression analysis were applied to construct a novel prognostic model based on ER stress-related DEGs. Next, we confirmed the predictive performance of this model with the GEO dataset and explored the potential biological functions by functional enrichment analysis. Finally, KIRC patients stratified by the prognostic model were assessed for tumor microenvironment (TME), immune infiltration, and immune checkpoints through single-sample Gene Set Enrichment Analysis (ssGSEA) and ESTIMATE analysis.

**Results:** We constructed a novel prognostic model, including eight ER stress-related DEGs, which could stratify two risk groups in KIRC. The prognostic model and a model-based nomogram could accurately predict the prognosis of KIRC patients. Functional enrichment analysis indicated several biological functions related to the progression of KIRC. The high-risk group showed higher levels of tumor infiltration by immune cells and higher immune scores.

**Conclusion:** In this study, we constructed a novel prognostic model based on eight ER stress-related genes for KIRC patients, which would help predict the prognosis of KIRC and provide a new orientation to further research studies on personalized immunotherapy in KIRC.

## Introduction

Renal cell carcinoma (RCC) has been considered one of the most common malignancies of the urinary system, with an increased incidence rate year by year (Znaor et al., 2015). Kidney renal clear cell carcinoma (KIRC) is the most representative histopathologic subtype of renal cell carcinoma, constituting approximately 75% of renal cell carcinoma (Moch et al., 2016; Wei et al., 2019). Although there have been advances in multiple therapeutic methods, including surgery, chemotherapy, and immunotherapy, the prognosis of KIRC patients remains a major clinical challenge because of the high incidence of recurrence and metastasis. As reported, the recurrence rate of KIRC after curative nephrectomy is approximately 20–30% within 5 years (Huang et al., 2021). More importantly, studies have shown that the 5-year overall survival rate is less than 10% in metastatic KIRC patients (Xing et al., 2020; Lamprou et al., 2021). Recently, immunotherapy has provided additional options for KIRC patients and brought new hope for the treatment of KIRC, such as immune checkpoint inhibitors (ICIs) targeting programmed death 1/programmed death ligand 1 (PD-1/PD-L1) and cytotoxic lymphocyte–associated antigen 4 (CTLA-4) (Motzer et al., 2015; Motzer et al., 2020). However, studies have demonstrated that only specific types of KIRC patients could respond to immunotherapy (George et al., 2019; Derosa et al., 2020). Moreover, various research studies have implied that prognostic models based on biomarkers could be used to assess the survival outcome in KIRC, but some models are more or less flawed (Bai et al., 2021; Sun et al., 2022). Therefore, as the poor prognosis and difficult treatment of KIRC, it is imperative to exploit novel reliable models to evaluate the clinical characteristics and provide prognostic indicators and clinical individualized treatments for KIRC.

The vital design of anticancer therapy depends on the identification and application of the weak points of cancer cells. As the largest organelle in cells, the endoplasmic reticulum (ER) is a major site of protein synthesis, processing, and transport (Hetz et al., 2020). ER stress is a relatively novel cellular unfolded protein response that maintains cell survival resulting from the accumulation of misfolded protein in the ER. A number of factors, including hypoxia, oxidative stress, metabolic stress, and nutrient depletion, can reduce the capacity of protein folding and induce ER stress (Hetz and Papa, 2018). ER stress is found to be involved in the progression of various cancers. Accumulating evidence has shown that ER stress has emerged as a crucial player in tumor progression, immunity, angiogenesis, and chemotherapy resistance (Harnoss et al., 2020; Gilardini Montani et al., 2021). As reported, ER stress can promote tumor progression and chemotherapy by regulating the metabolic state of tumor cells in gliomas (Zhang et al., 2021). Additionally, ER stress can regulate the role of infiltrating immune cells by inducing tumor cell escape from immunological surveillance. For example, ER stress stimulates a series of inflammatory factors, including IL-23 and IL-6, in macrophages, thereby promoting tumor progression and metastasis through modifying the immune characteristics of tumor cells (Cao et al., 2016). ER stress is induced by three important members of transmembrane ER sensors: protein kinase R-like ER kinase (PERK), inositol requiring enzyme 1 (IRE1), and activating transcription factor 6 (ATF6). As reported, the IRE1/XBP-1 pathway promotes tumor progression in chronic lymphocytic leukemia and breast cancer (Chen et al., 2014; Tang et al., 2014). In addition, STF-083010, as the effective inhibitor of IRE1, suppresses the progression of solid tumors by reducing the T-cell expression of PD-1 (Zhan et al., 2019). Therefore, considering the crucial role of ER stress in tumor progression, ER stress and related genes may become the potential prognostic biomarkers and important targets in the treatment of KIRC.

**FIGURE 1:**
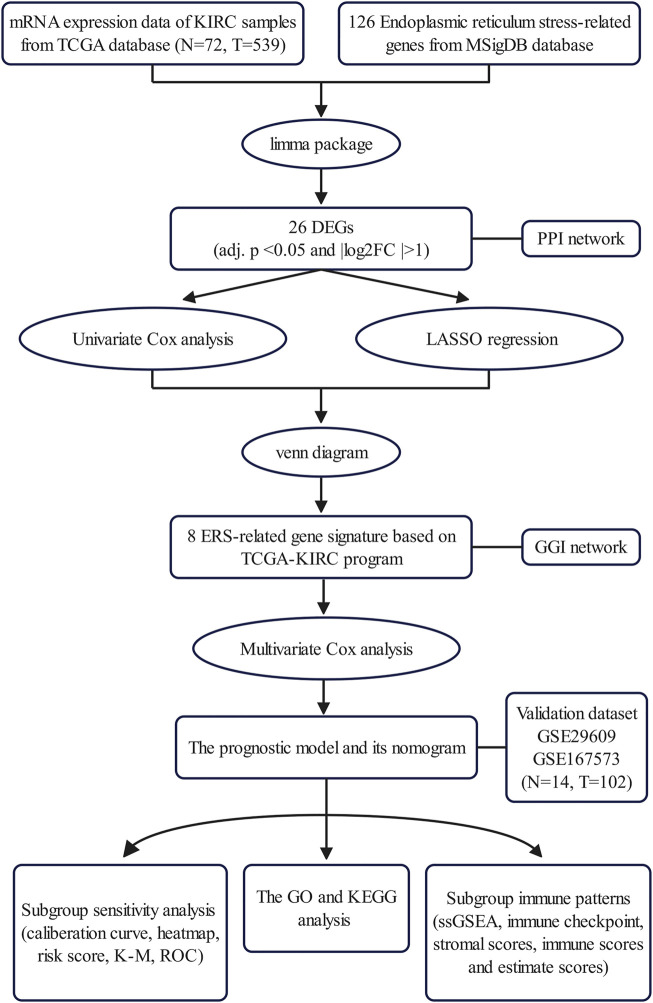
Outline of the analysis process.

In this study, to explore and evaluate the potential clinical value of ER stress in KIRC, we performed a series of bioinformatics analyses to construct a novel prognostic ER stress-related gene model through using The Cancer Genome Atlas (TCGA) database and confirmed the predictive performance of the prognostic model in the Gene Expression Omnibus (GEO) dataset. Hence, the prognostic model and a model-based nomogram could accurately predict the prognosis of KIRC patients. Additionally, we explored the potential biological functions by functional enrichment analysis. Finally, this novel prognostic model could provide a theoretical basis for tumor microenvironment (TME), immune infiltration, and immune checkpoints in KIRC (Figure 1).

## Materials and methods

### Data collection

The mRNA sequencing data and clinical features of kidney renal clear cell carcinoma patients were acquired from the TCGA-KIRC program (https://portal.gdc.cancer.gov) to build the ER stress-related prognostic training model (Liu et al., 2018). The TCGA-KIRC program includes 72 normal samples and 539 carcinoma samples. GSE29609 and GSE167573 were selected as the verification cohorts. The corresponding expression data and follow-up files were downloaded from the GEO (https://www.ncbi.nlm.nih.gov/geo) database. GSE29609 and GSE167573 include 14 normal samples and 102 carcinoma samples. Samples lacking reliable clinicopathological or survival information were excluded from further analysis.

The ER stress-related pathways were collected from the Molecular Signatures Database v.7.5.1 (MSigDB; https://www.gsea-msigdb.org/gsea/msigdb). In total, 126 ER stress-related genes were mined from the 13 pathways in Reactome and Gene Ontology (GO) databases. GEPIA2 (http://gepia2.cancer-pku.cn/#index) was used to draw a survival map of 33 cancer types in order to visualize the correlation between the expression of these 126 genes and the overall survival time of different tumors.

### Screening of ER stress-related differentially expressed genes

To explore the differentially expressed genes (DEGs) among normal and KIRC specimens, we analyzed the expression data on 126 ER stress-related genes from the TCGA-KIRC program *via* the R package “limma”. We used adj. *p* < 0.05 and |log2FoldChange |>1 as the threshold and obtained 26 DEGs. To visualize expression data, the heatmap, volcano plot, and bar charts were plotted *via* the R packages “ComplexHeatmap” and “ggplot2”.

### Biological function and genetic mutation landscape of ER stress-related DEGs

The Search Tool for the Retrieval of Interacting Genes (STRING; http://string-db.org) database was used to depict the protein–protein interaction (PPI) network among these DEGs with a combined score of >0.15. Cytoscape (version 3.8.2) and its plugins, BiNGO and MCODE, were utilized to show gene ontology categories and hub genes.The cBioPortal (http://www.cbioportal.org) helped illustrate the mutation spectrum in KIRC patients.

### Screening of prognosis-related genes

Univariate Cox regression analysis was employed to identify prognosis-related genes of KIRC patients from TCGA database *via* the R packages “survminer” and “survival”. Next, the least absolute shrinkage and selection operator (LASSO) regression was applied to screen genes which were significantly related to survival time *via* the R packages “glmnet” and “survival”. Then, we plotted a Venn diagram to show the intersection between genes derived from univariate Cox regression and from lambda.min of LASSO analysis. We also depicted the gene–gene interaction (GGI) network *via* GeneMANIA (https://genemania.org).

### Construction of a prognostic model based on ER stress-related genes

We calculated the risk scores of each sample. The formula was as follows: Σi coefficient (gene_i) × expression (gene_i). So far, the samples were divided into two risk groups, according to the median risk score. In addition, the Kaplan–Meier (K-M) curve, the time-dependent receiver operating characteristic (ROC) curve, the heatmap, and the forest plot using both univariate and multivariate Cox regression analyses were plotted to illustrate the distribution and survival status of KIRC patients in two risk subgroups.

### Establishment and verification of the nomogram

A nomogram was plotted to predict the survival of KIRC patients *via* the R packages “rms” and “survival”. It covered different variables, including age, gender, race, TNM stage, histological grade, tumor laterality, and risk score. The 1-year, 3-year, and 5-year calibration curves were plotted to validate the veracity of the nomogram *via* the R package “rms”. Moreover, the samples from two GEO series were combined as a verification cohort to validate the efficiency of the constructed prognostic model.

### Functional enrichment exploration

GO analysis and Kyoto Encyclopedia of Genes and Genomes (KEGG) analysis were applied to reveal underlying pathways *via* the R package “clusterProfiler”. The corresponding chord diagram was plotted *via* the R package “GOplot”.

### Immune pattern analysis of subgroups in KIRC

The infiltrating fractions of immune cells were calculated with a single-sample Gene Set Enrichment Analysis (ssGSEA) algorithm *via* the R package “GSVA” (Hanzelmann et al., 2013). Then, the infiltrating levels of immune cells and immune checkpoints were compared in the different risk score groups. In addition, we calculated the stromal scores, immune scores, and estimate scores of KIRC patients *via* the R package “ESTIMATE” (Liu et al., 2020).

### Statistical analysis

R software (version 4.1.1) and its relevant packages were applied to process, analyze, and present the data. IBM SSPS software (SPSS Inc., Chicago, IL, United States) was also a requisite. A *p*-value < 0.05 was deemed as statistically valuable.

## Result

### Distinction of ER stress-related DEGs in KIRC

**FIGURE 2:**
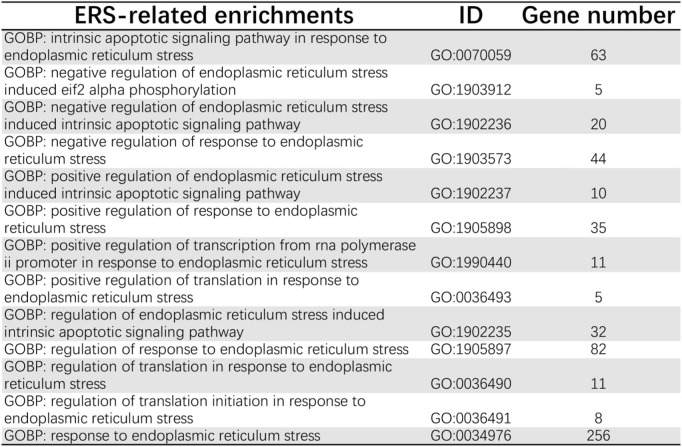
ER stress-related pathways from MSigDB.

**FIGURE 3:**
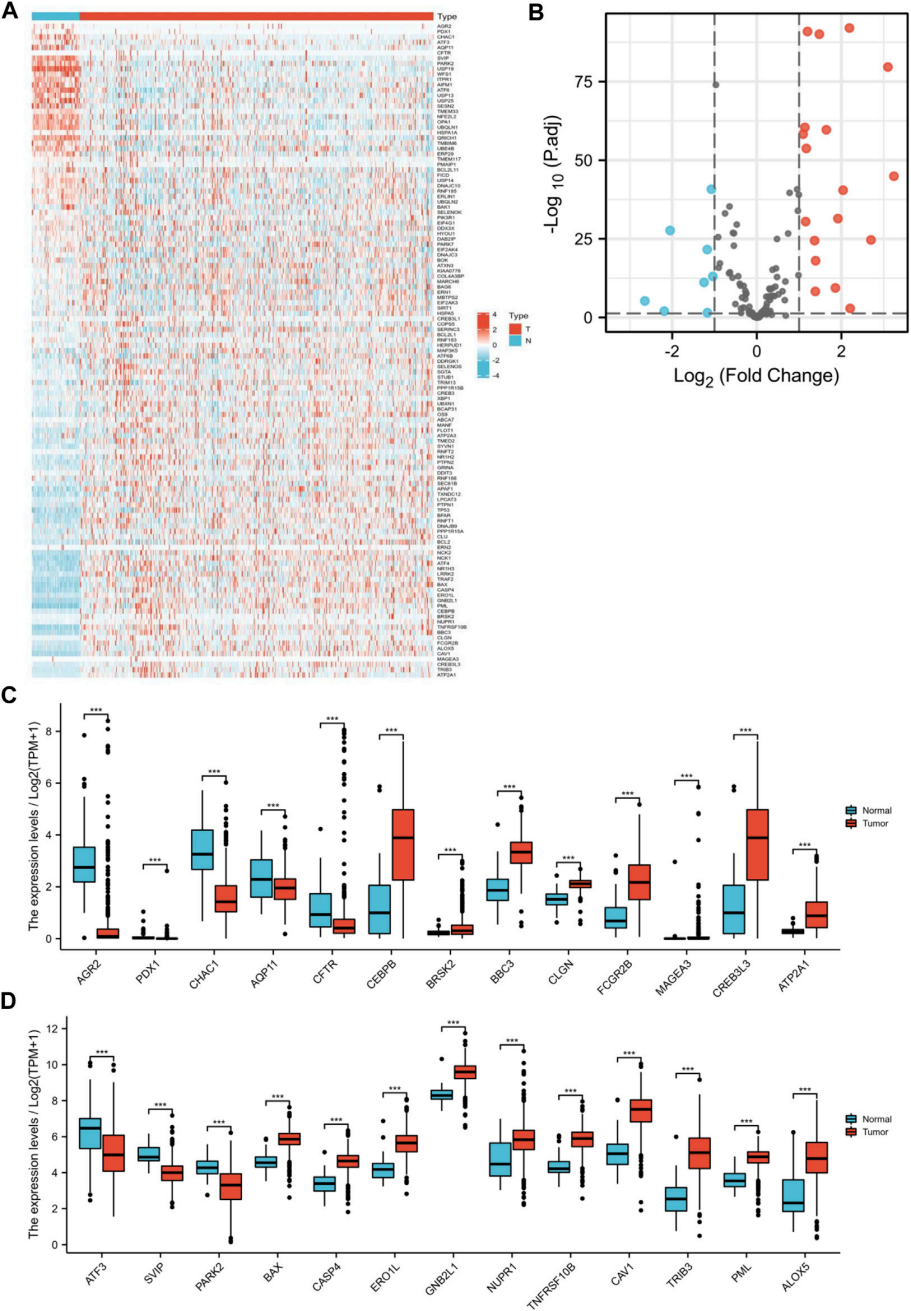
Distinction of ER stress-related DEGs in KIRC. ER stress-related DEGs were identified between KIRC and normal specimens in TCGA dataset with the cutoff values of |log_2_FC| > 1. **(A)** Heat map visualized upregulated and downregulated ER stress-related genes in tumor specimens (T) and normal specimens (N). Red means upregulated genes, and blue means downregulated genes. **(B)** Volcano map visualized upregulated and downregulated ER stress-related genes. Red means upregulated genes, blue means downregulated genes, and gray means nonsignificant genes. **(C,D)** Expression of 26 ER stress-related genes between tumor specimens (T) and normal specimens (N) (***p < 0.001).

First, we acquired 126 ER stress-related pathways from MSigDB (Figure 2). Next, we identified the different expression levels of 126 ER stress-related genes in 539 tumor specimens and 72 normal specimens with TCGA dataset (Figure 3A). Additionally, we evaluated the correlation between the expression levels of 126 ER stress-related genes and the overall survival time of different tumors through GEPIA2. As shown in Supplementary Figure S1, we identified the different hazard ratios (HRs) of 126 ER stress-related genes in different tumors. Then, under the cutoff values of |log_2_FoldChange| > 1 and *p* < 0.05, a total of 26 DEGs were found; we identified that 18 ER stress-related genes displayed markedly high expression and eight ER stress-related genes displayed markedly lower expression in KIRC than normal tissues in TCGA dataset (Figures 3B–D).

### Biological function and genetic mutation landscape of ER stress-related DEGs in KIRC

**FIGURE 4:**
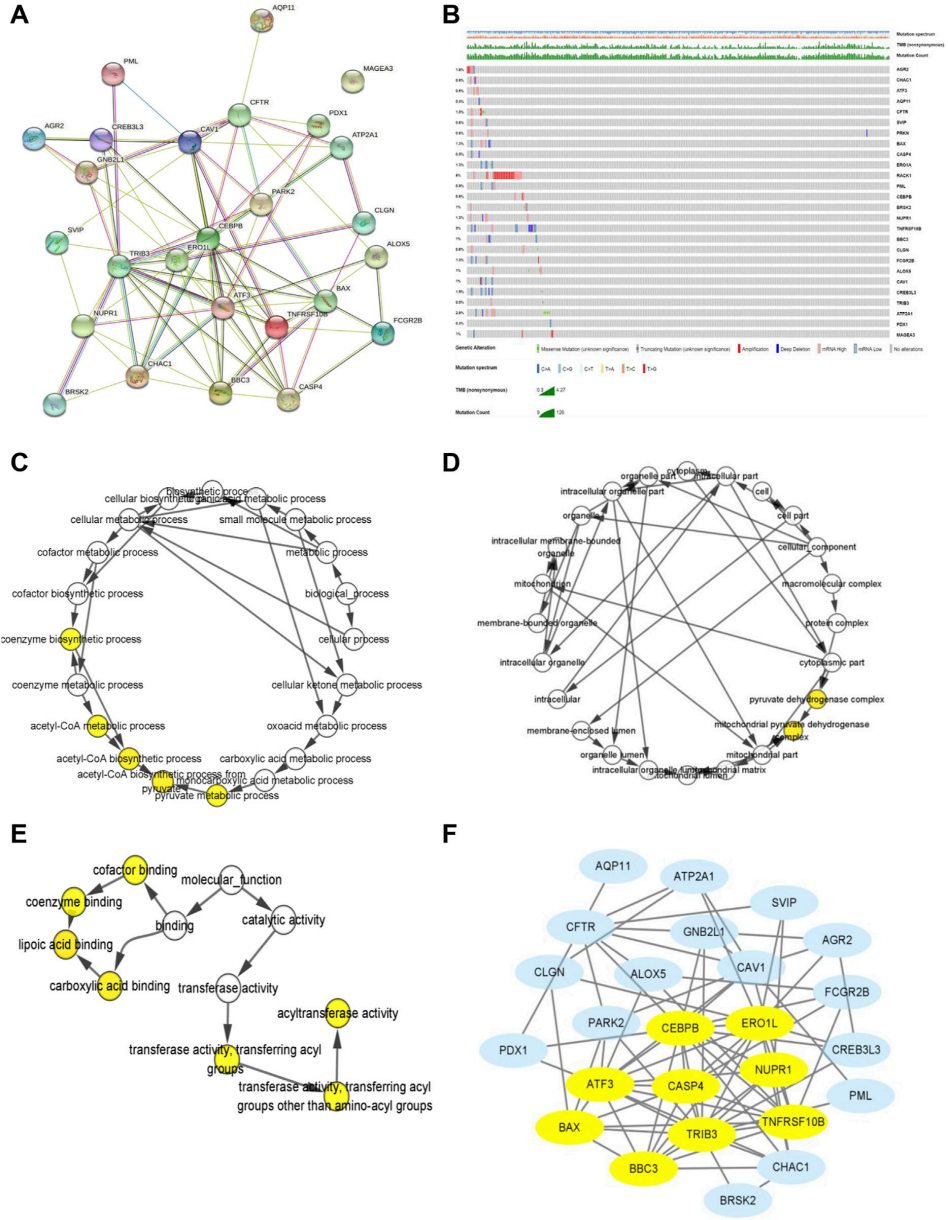
Biological function and genetic mutation landscape of ER stress-related DEGs in KIRC. **(A)** PPI network acquired from the STRING website of ER stress-related DEGs. **(B)** Main types of genetic mutations of ER stress-related DEGs. **(C–E)** Biological processes, cellular components, and molecular functions of ER stress-related DEGs. **(F)** Relationship among the protein expression of 26 ER stress-related DEGs by using the MCODE plugin. Yellow means hub genes.

A PPI analysis by the STRING website was performed to illustrate the interactivity of DEGs (Figure 4A). Next, we explored the genetic mutations of these DEGs in KIRC. As shown in Figure 4B, the main types of genetic mutations were missense, truncating, amplification, deep deletion, and mRNA high. Additionally, the results suggested that RCAK1 (8%) was the gene with the highest mutation incidence, followed by TNFRSF10B (3%), among the 26 ER stress-related genes. Furthermore, functional enrichment analyses were conducted to demonstrate the biological process, cellular component, and molecular function involving the 26 ER stress-related DEGs. ER stress-related DEGs were prominently enriched in the biological processes of coenzyme biosynthetic process, acetyl-CoA metabolic process, acetyl-CoA biosynthetic process, and pyruvate metabolic process (Figure 4C). Meanwhile, cellular components of the pyruvate dehydrogenase complex and mitochondrial pyruvate dehydrogenase complex were markedly modulated by these ER stress-related genes (Figure 4D). These ER stress-related DEGs possessed the molecular functions of cofactor binding, coenzyme binding, lipoic acid binding, carboxylic acid binding, acyltransferase activity, and transferase activity (Figure 4E). Then, we further identified *CEBPB*, *ERO1L*, *ATF3*, *BAX*, *BBC3*, *TRIB3*, *TNFRSF10B*, *NUPR1*, and *CASP4* were hub genes by MCODE analysis (Figure 4F). Collectively, these results demonstrated the potential role of these ER stress-related DEGs in KIRC tumorigenesis.

### Construction of the prognostic model based on ER stress-related DEGs

**FIGURE 5:**
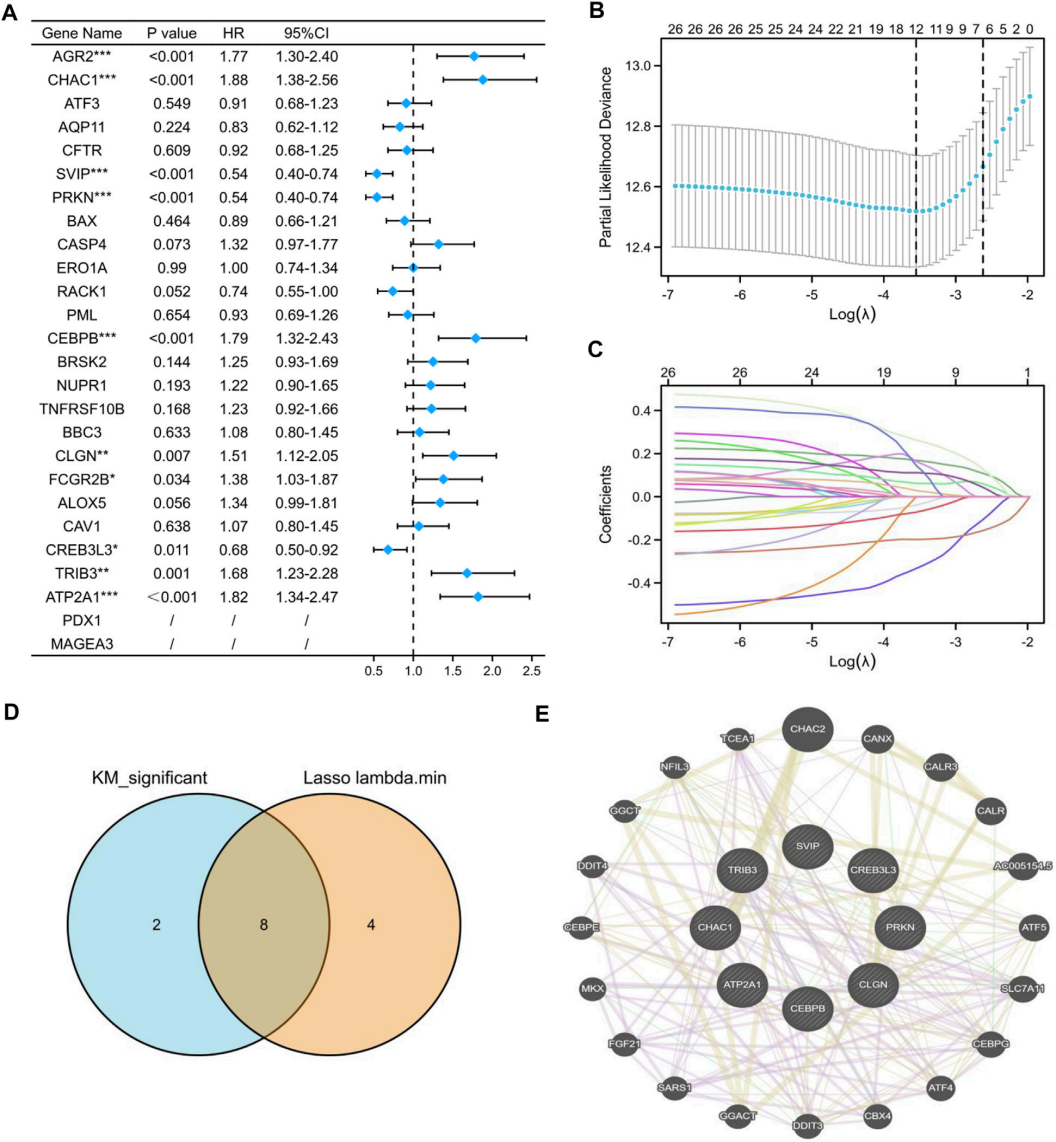
Construction of the prognostic model based on ER stress-related DEGs. **(A)** Univariate Cox regression analysis of 26 ER stress-related DEGs. **(B,C)** LASSO coefficient profiles of 26 ER stress-related DEGs. **(D)** Venn diagram visualized the intersecting genes between the K-M-related DEGs and lambda minimum genes. **(E)** GGI analysis by the GeneMANIA website among candidate ER stress-related DEGs. (*p < 0.05, **p < 0.01, and ***p < 0.001).

To construct an ER stress-related prognostic model, we further screened 26 candidate prognostic ER stress-related DEGs by univariate Cox regression analysis. As shown in Figure 5A, among the 26 prognostic ER stress-related DEGs, *AGR2*, *CHAC1*, *CEBPB*, *CLGN*, *FCGR2B*, *TRIB3*, and *ATP2A1* were regarded as high-risk genes based on their HRs, whereas *SVIP*, *PRKN*, and *CREB3L3* were regarded as low-risk genes. Subsequently, through a LASSO Cox regression analysis with 10-fold cross-validation to identify the optimal lambda value (*λ*) which came from the minimum partial likelihood deviance, *CHAC1*, *ATF3*, *SVIP*, *PRKN*, *BAX*, *CASP4*, *CEBPB*, *CLGN*, *CREB3L3*, *TRIB3*, *ATP2A1*, and *PDX1* were significantly associated with the prognosis of KIRC (Figures 5B,C). After intersecting the aforementioned K-M related DEGs and lambda minimum genes, we obtained eight ER stress-related DEGs as candidate prognostic model-related genes (Figure 5D). In addition, to reveal the latent intermediate genes among these eight genes from three aspects, including co-expression, shared protein domains, and genetic interactions, we performed a GGI analysis by using the GENEMANIA website (Figure 5E).

**FIGURE 6:**
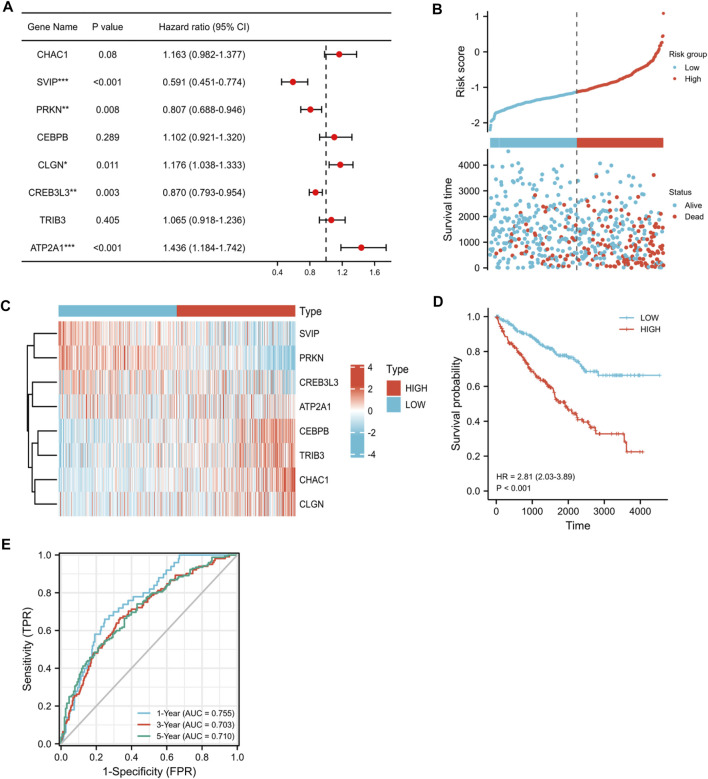
Correlation between the eight candidate ER stress-related DEGs and the prognosis of KIRC. **(A)** Multivariate Cox regression analysis of eight candidate ER stress-related DEGs. **(B)** Ranked dot and scatter plots showing the risk score distribution and patient survival status. **(C)** Heat map visualized upregulated and downregulated eight candidate ER stress-related DEGs between two risk groups in tumor specimens. Red means upregulated genes, and blue means downregulated genes. **(D)** K-M analysis of the OS between the two risk groups. **(E)** ROC curves to predict the sensitivity and specificity of 1-, 3-, and 5-year survival, according to the risk score. (*p < 0.05, **p < 0.01, and ***p < 0.001).

To further verify the correlation between the eight candidate ER stress-related DEGs and the prognosis of KIRC, we screened the eight candidate ER stress-related DEGs by multivariate Cox regression analysis. Among the eight genes, *CHAC1*, *CEBPB*, *CLGN*, *TRIB3*, and *ATP2A1* were regarded as high-risk genes, whereas *SVIP*, *PRKN*, and *CREB3L3* were regarded as low-risk genes (Figure 6A). Ultimately, according to our results, we developed a prognostic model, which contained *CHAC1*, *SVIP*, *PRKN*, *CEBPB*, *CLGN*, *CREB3L3*, *TRIB3*, and *ATP2A1*. Then, the risk scores were computed on the basis of the normalized expression of eight prognostic ER stress-related genes and their regression coefficients: risk score = (0.16891800 * CHAC1 expression) + (-0.33279463 * SVIP expression) + (-0.19868751 * PRKN expression) + (0.06170303 * CEBPB expression) + (0.11164955 * CLGN expression) + (-0.07175666 * CREB3L3 expression) + (0.02883605 * TRIB3 expression) + (0.26298195 * ATP2A1 expression). Therefore, the patients were separated into two risk groups (low and high) on the foundation of the median risk score. As shown in Figure 6B, the mortality of the high-risk group was higher than that of the low-risk group. Moreover, our result demonstrated the assignation of the eight ER stress-related DEG expressions in tumor specimens in the subgroups of the risk score (Figure 6C). In addition, K-M curves indicated that the patients in the high-risk group had worse overall survival than the patients in the low-risk group (*p* < 0.001, Figure 6D). To assess the accuracy of the prognostic model, we also performed a time-dependent ROC curve. As shown in Figure 6E, the area under the ROC curve (AUC) was 0.755 for 1-year overall survival (OS), 0.703 for 3-year OS, and 0.710 for 5-year OS, indicating that this eight-gene prognostic model performed well as a predictor of OS.

### Independent prognostic value and clinical utility of the prognostic model

**FIGURE 7:**
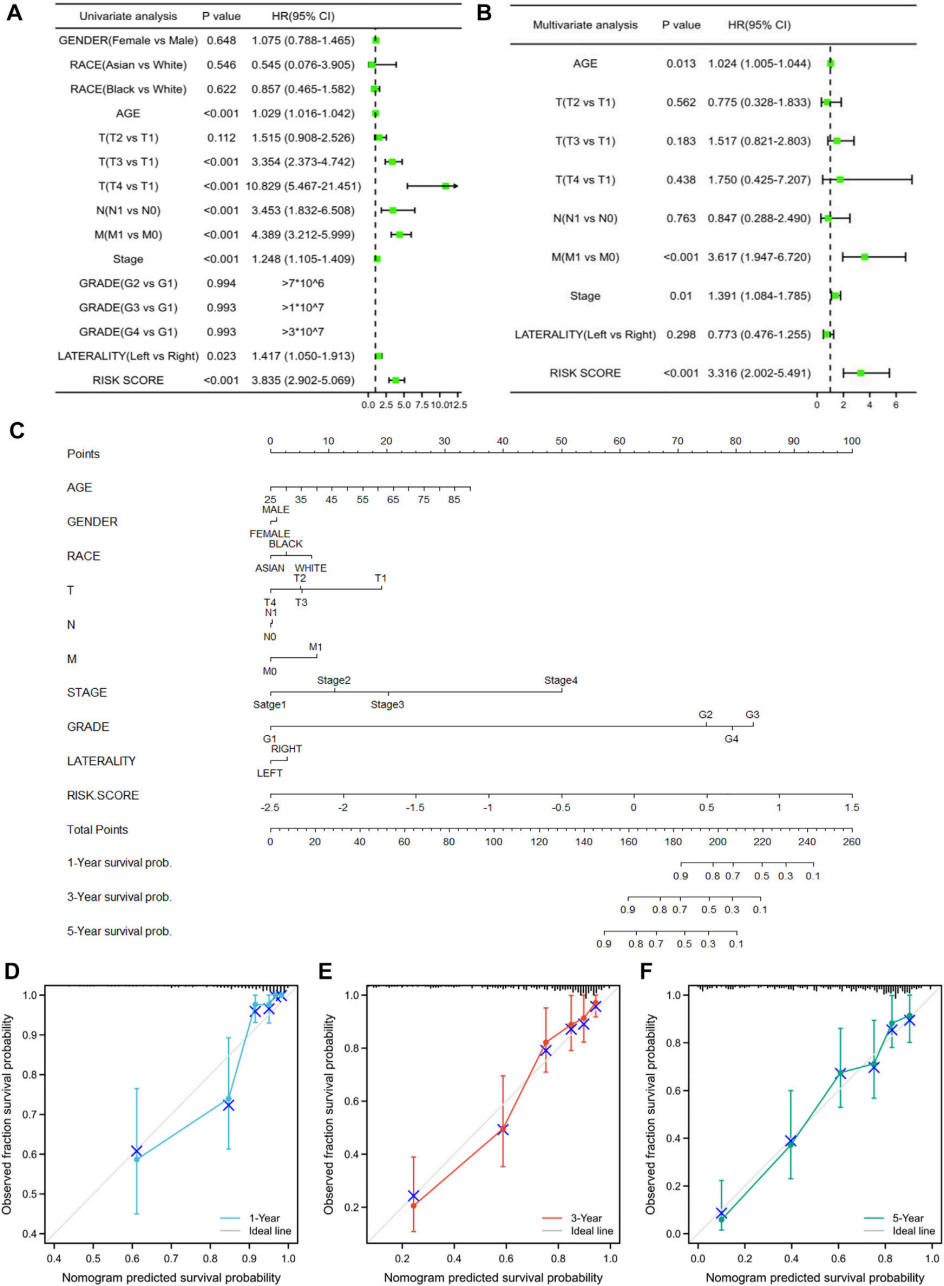
Independent prognostic value and clinical utility of the prognostic model. **(A,B)** Univariate and multivariate Cox regression analyses for investigating the association of the ER stress-related gene model and conventional clinical factors with KIRC prognosis. **(C)** Prognostic nomogram based on the ER stress-related model for prediction of 1-, 3-, and 5-year survival rates. **(D–F)** Calibration plots of the nomogram at 1-, 3-, and 5-year survival.

We then performed univariate and multivariate Cox regression analyses to examine whether the prognostic model was an independent prognostic value for other clinical features. The univariate Cox regression analysis showed that age, T3-stage, T4-stage, N-stage, M-stage, and risk score were marked correlated with KIRC prognosis (Figure 7A). Following multivariate Cox regression analysis, M-stage and risk score acted as independent risk factors of KIRC (Figure 7B). Furthermore, to provide clinicians with a more accurate and reliable prognostic model, we performed a nomogram by integrating the risk score and other clinical parameters, indicating 1-, 3-, and 5-year OS for KIRC patients (Figure 7C). Meanwhile, the calibration curves of the constructed nomogram presented great accuracy between predicted values and actual observations (Figures 7D–F).

### Validation of the prognostic ER stress-related gene model

**FIGURE 8:**
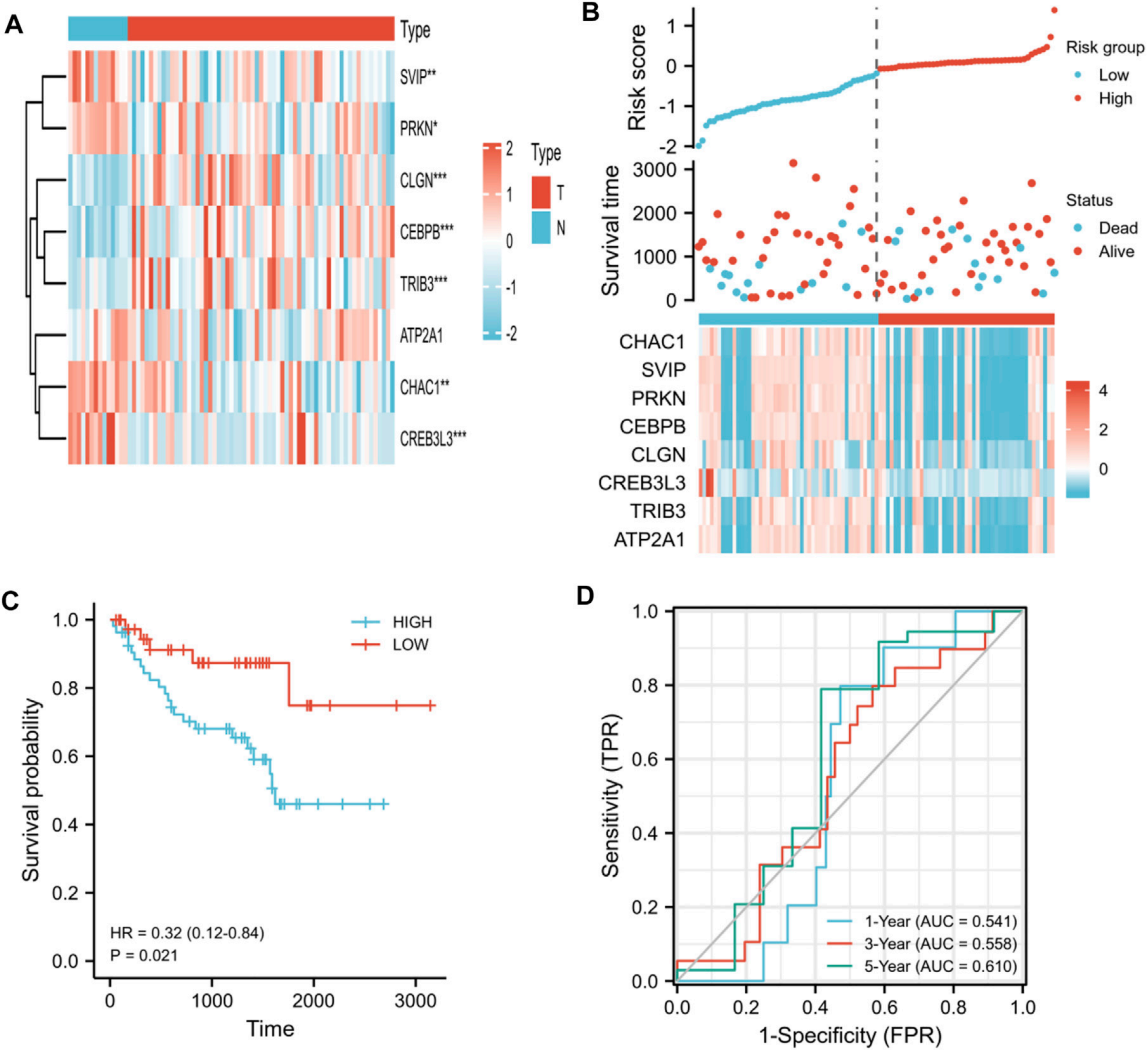
Validation of the prognostic ER stress-related gene model by using the patients from GSE29609 and GSE167573 datasets. **(A)** Heat map visualized differential expression of eight ER stress-related genes in tumor specimens (T) and normal specimens (N). Red means upregulated genes, and blue means downregulated genes. **(B)** Ranked dot and scatter plots showing the risk score distribution and patient survival status. **(C)** K-M analysis of the OS between the two risk groups. **(D)** ROC curves to predict the sensitivity and specificity of 1-, 3-, and 5-year survival according to the risk score. (*p < 0.05, **p < 0.01, and ***p < 0.001).

To validate the prognostic model we constructed, we further evaluated this model in the testing set by using the patients from GSE29609 and GSE167573 datasets. First, we evaluated the different expression levels of the eight prognostic ER stress-related genes in the tumor specimens and normal specimens. Our results suggested that *CHAC1*, *CEBPB*, *CLGN*, *TRIB3*, and *ATP2A1* had high expression in KIRC; on the contrary, *SVIP*, *PRKN*, and *CREB3L3* had low expression (Figure 8A). Next, based on the aforementioned median risk score, the patients were separated into high-risk groups and low-risk groups. As shown in Figure 8B, the mortality of the high-risk group was higher than that of the low-risk group; meanwhile, the assignation of the eight ER stress-related gene expressions in tumor specimens were plotted in the subgroups of the risk score. The K-M curves indicated that the patients in the high-risk group had worse overall survival than the patients in the low-risk group (*p* < 0.001, Figure 8C). In addition, the AUC was 0.541 for 1-year OS, 0.558 for 3-year OS, and 0.610 for 5-year OS (Figure 8D). These results collectively indicated that the prognostic model we constructed performed well as a predictor of OS in KIRC.

### Functional analysis of DEGs based on the prognostic model

**FIGURE 9:**
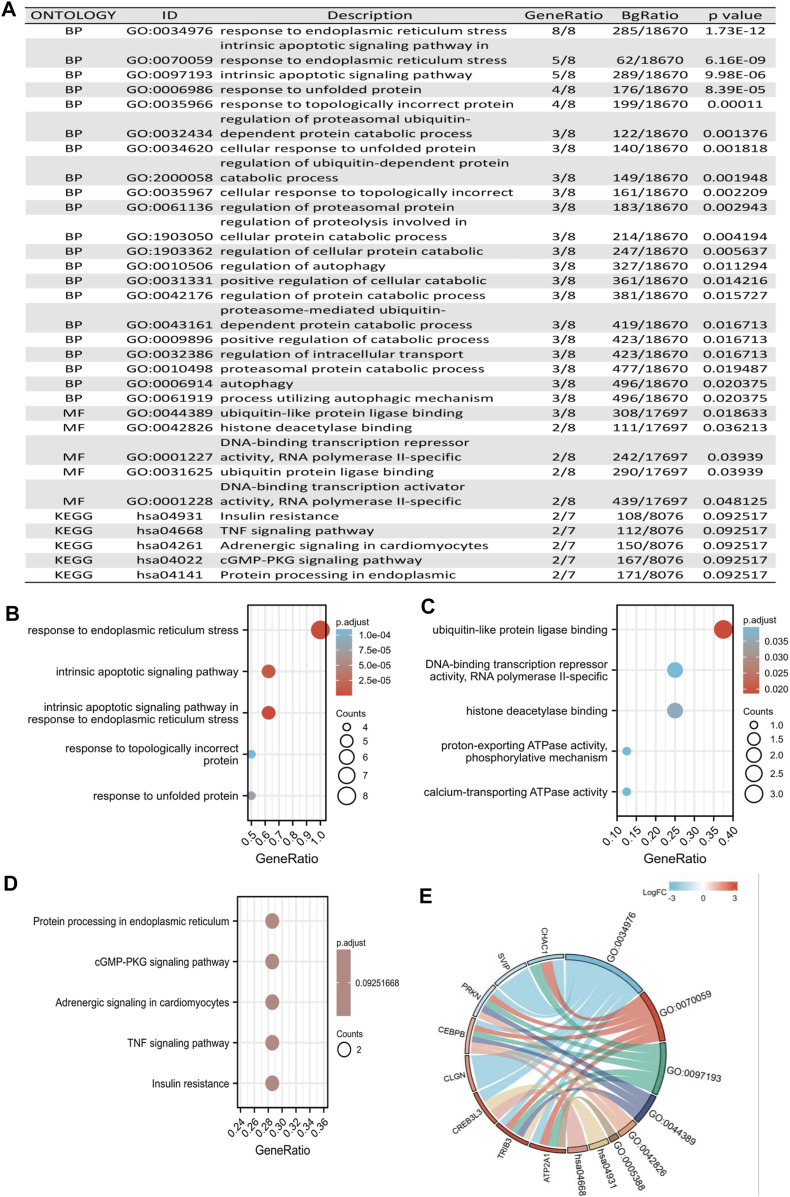
Functional analysis of DEGs based on the prognostic model. **(A)** Potential biological functions between the two risk groups in KIRC. **(B,C)** GO enrichment analysis of DEGs based on a prognostic model, including biological processes and molecular function. **(D)** KEGG pathway enrichment analysis of DEGs based on the prognostic model. **(E)** Chord diagram visualized the correlation between eight ER stress-related DEGs and potential biological functions.

To explore the potential biological functions between the two risk groups in KIRC, we identified the GO terms and KEGG of the eight ER stress-related DEGs (Figure 9A). The BP GO analysis showed that differentially expressed genes were mainly enriched in the response to ER stress, intrinsic apoptotic signaling pathway, intrinsic apoptotic signaling pathway in response to ER stress, and topologically incorrect protein (Figure 9B). For MF analysis, differentially expressed genes were significantly enriched in ubiquitin-like protein ligase binding, DNA-binding transcription repressor activity, RNA polymerase II-specific, histone deacetylase binding, and calcium-transporting ATPase activity (Figure 9C). However, for CC analysis, there was no term that was enriched. In addition, according to the KEGG pathway analysis, differentially expressed genes were primarily correlated with protein processing in the ER, cGMP-PKG signaling pathway, adrenergic signaling in cardiomyocytes, TNF signaling pathway, and insulin resistance (Figure 9D). Furthermore, we identified the correlation between eight ER stress-related DEGs and potential biological functions (Figure 9E).

### Assessment of the tumor microenvironment and immune checkpoints

**FIGURE 10:**
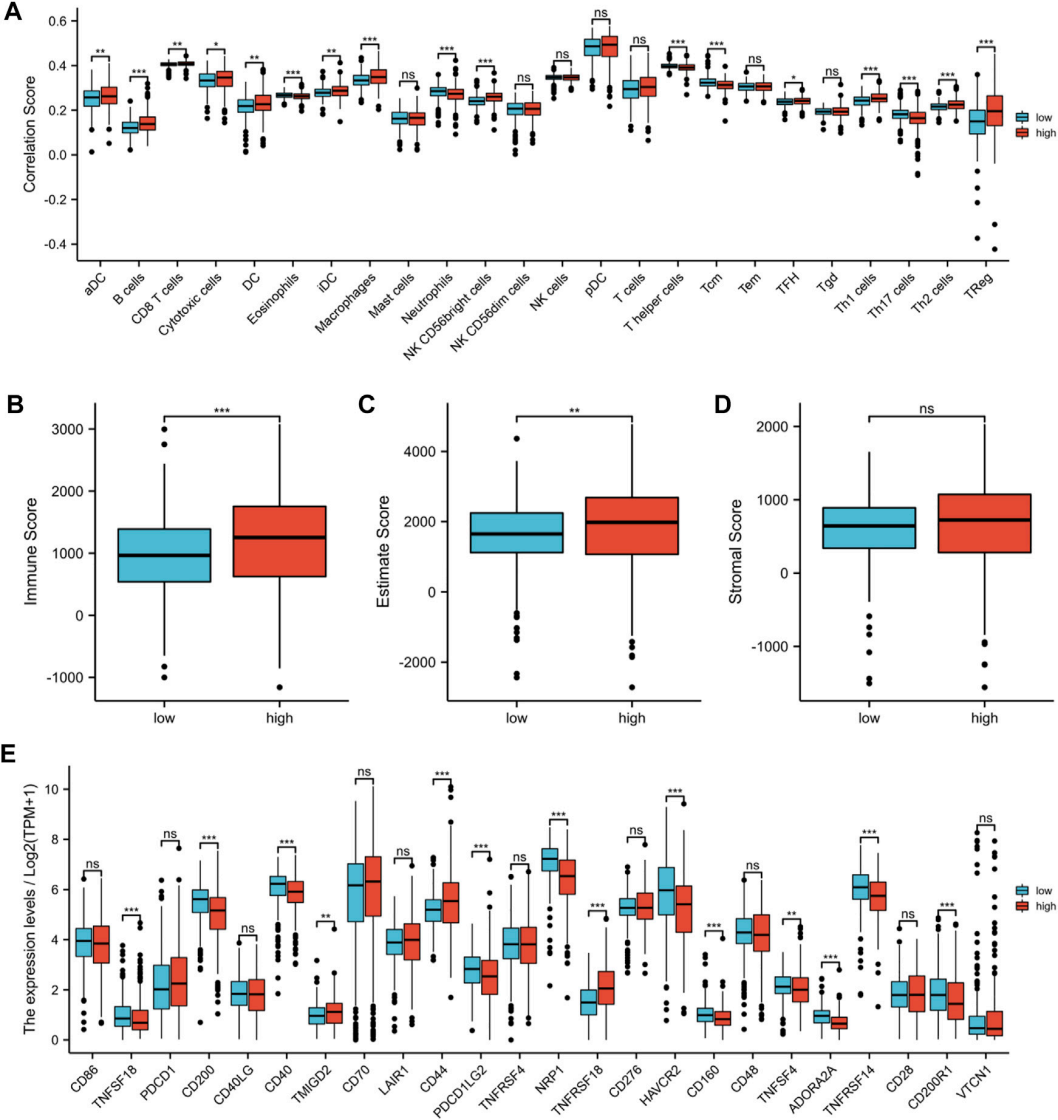
Assessment of the tumor microenvironment and immune checkpoints between two risk groups. **(A)** Infiltration levels of 24 immune cell types between high-risk and low-risk groups. **(B)** Immune score between high-risk and low-risk groups. **(C)** ESTIMATE score between high-risk and low-risk groups. **(D)** Stromal score between high-risk and low-risk groups. **(E)** Expression of immune checkpoints between high-risk and low-risk groups. (*p < 0.05, **p < 0.01, and ***p < 0.001).

Recent research studies had revealed that the tumor immune microenvironment was significantly correlated with malignant behavior; therefore, we assessed the unique features of the TME to distinguish between the two risk groups in KIRC. As shown in Figure 10A, the abundance of activated DC cells, B cells, CD8^+^ T cells, cytotoxic cells, DC cells, immature DC cells, macrophages, NK CD56 bright cells, TFH cells, Th1 cells, Th2 cells, and Treg cells in the high-risk group was significantly higher, whereas the abundance of eosinophils, neutrophils, T helper cells, Tcm cells, and Th17 cells was opposite. Then, we performed an ESTIMATE algorithm to further evaluate the correlation between risk score and immune cell infiltration. The immune score and the ESTIMATE score of the high-risk group were significantly higher (Figures 10B,C). However, there was no statistical difference in the stromal score between the two risk groups, but the high-risk group had a trend of higher stromal score (Figure 10D). In addition, we evaluated the correlation between the risk score and 24 immune checkpoints. Among the 24 immune checkpoints, 14 immune checkpoints were discrepantly represented in the two risk groups, such as TNFSF18, CD40, and CD44 (Figure 10E).

## Discussion

KIRC is a common and highly heterogeneous malignant tumor that develops through multiple complex biological processes. Due to the high metastasis and recurrence rate of KIRC, the efficacy of traditional treatment is limited, and the prognosis of KIRC patients is dismal. Thus, novel prognostic biomarkers and valid therapeutic targets are urgently needed. ER is the most important intracellular organelle for protein synthesis and cellular homeostasis, which is involved in the regulation of various signaling pathways. A specific state named “ER stress” will be triggered when ER homeostasis is disrupted by a number of intrinsic or extrinsic factors, such as hypoxia, oxidative stress, metabolic stress, and nutrient depletion. In fact, accumulating evidence has demonstrated the inevitable association between ER stress and the development of multiple cancers, including KIRC (Chen and Cubillos-Ruiz, 2021; Varone et al., 2021). Recently, according to numerous research studies, multiple gene models, which are applied to predict outcomes and therapeutic effect, seem to have high credibility (Divate et al., 2022; Zhai et al., 2022). However, most of the research studies focus on the effect of ER stress in cancer progression and metastasis, and few studies have illustrated the prognostic value of ER stress-related genes in cancers, especially in KIRC.

Hence, in the current study, we systematically evaluated the expression of 126 ER stress-related genes between KIRC specimens and normal specimens based on public databases and identified that 26 genes were differentially expressed in KIRC. Then, we performed PPI analysis and functional enrichment analyses to explore the potential biological function of 26 ER stress-related DEGs. Meanwhile, according to the hub genes by MCODE analysis, we further revealed the potential role of ER stress-related genes in KIRC tumorigenesis. Finally, an eight ER stress-related prognostic model was constructed by LASSO and univariate Cox regression analyses. To further explore the prognostic value and clinical significance, we also performed survival and ROC analysis to validate the predictive performance of this novel prognostic model. Moreover, subgroup analyses stratified by clinical features also evidenced that KIRC patients with low-risk scores had better overall survival than those with high-risk scores. Altogether, these results indicated that the eight ER stress-related gene prognostic model had a significant potential in clinical application of KIRC patients.

In the eight ER stress-related DEGs which were significantly associated with the prognostic signature, *CHAC1*, *CEPBP*, *CLGN*, *TRIB3*, and *ATP2A1* were risk factors, while *SVIP*, *PRKN*, and *CREB3L3* were protective factors. Glutathione (GSH) acted as a reactive oxygen species (ROS) scavenger and was significantly associated with the induction of ER stress (Proneth and Conrad, 2019). *CHAC1* was a novel proapoptotic member of ER stress-related genes and possessed *γ*-glutamyl cyclotransferase activity, thus regulating the degradation of GSH (Cui et al., 2021). Previous research studies suggested that *CHAC1* was correlated with a high risk of metastasis and poor prognosis and exerted a harmful influence on clinical outcomes in uveal melanoma (Liu et al., 2019). As reported, tumors should adapt to the ER stress mechanisms which contained the unfolded protein response (UPR) and the ER-associated degradation (ERAD) to keep protein homeostasis if they were to survive and grow. Thus, ERAD ensured protein quantity and quality through degrading misfolded or unassembled proteins by the ubiquitin–proteasome system (Vembar and Brodsky, 2008). Mounting research studies suggested that small VCP interacting protein (SVIP), an endogenous inhibitor of ERAD, acted as a tumor feature, and its recovery after epigenetic silencing was correlated with increased ER stress and tumor growth inhibition. Meanwhile, this research also demonstrated that cancer cells harboring DNA methylation-associated loss of SVIP could obtain the cellular energy for their survival mainly through glucose and aerobic glycolysis, while SVIP restoration could promote the use of the homeostatic mitochondrial respiration (Llinas-Arias et al., 2019). PRKN, also called PARK2, is a cytosolic E3 ubiquitin ligase and was first shown to be involved in Parkinson’s disease (Kitada et al., 1998). To date, evidence indicated that PRKN is a tumor suppressor. As reported, downregulation of PRKN had been associated with ovarian, colorectal, and cervical cancers (Song et al., 2013; Klimczak et al., 2016; Bhat et al., 2019). CCAAT/enhancer binding protein beta (CEBPB), as a family of transcription factors of the basic leucine zipper (bZIP) class, is bound to DNA as homodimers and heterodimers. CEBPB could upregulate the expression of various target genes and was involved in numerous metabolic processes. Additionally, CEBPB could also regulate sequences of genes which were correlated with inflammatory response or ER stress (Akira et al., 1990; Meir et al., 2010). Recent studies had revealed that the expression of CEBPB was high in ovarian cancer, breast cancer, and colorectal cancer (Hungness et al., 2002). Calmegin (CLGN), a vital component for the membrane transport of target proteins, had been shown to be positively correlated with the progression of breast cancer (Ozkaya et al., 2017). CREB3L3, which was an ER stress-related transcription factor, had been proven to exert a tumor suppressor role in various cancers. For instance, CREB3L3 was associated with the proliferation and prognosis of hepatocellular carcinoma by regulating PI3K/Akt and AMPK signaling pathways (Vecchi et al., 2014). Additionally, CREB3L3 could initiate the human innate immune system by regulating the M2 marker gene expression in macrophages (Luan et al., 2015). However, the relationship between CREB3L3 and KIRC had not been investigated in detail. TRIB3 was a member of the pseudokinase tribbles family that played an important role in ER stress. Recent research studies evidenced that the expression of TRIB3 could be upregulated in a number of cancers, such as breast cancer, colorectal cancer, and lung cancer (Wennemers et al., 2011; Yu et al., 2019; Yu et al., 2020). Meanwhile, TRIB3 was involved in tumor progression and was related to a poor prognosis. ATP2A1, also called SERCA1, exerted a critical role in modulating ER Ca^2+^ dynamics. Several studies had shown that ATP2A1 was associated with apoptosis and immune responses in various cancers (Chemaly et al., 2018). For instance, a decrease in the ATP2A1 activity was associated with the upregulation of PD-L1 under glutamine-limited conditions (Byun et al., 2020).

Previous research studies have revealed that ER stress could promote cancer cells to evade immunity and facilitate metastasis. Moreover, ER stress was highly correlated with the TME. As reported, immune cells and stromal cells are the critical elements of the TME, and immune score and stromal score are correlated with the clinical features and prognosis in KIRC. Therefore, we calculated these scores by using the ESTIMATE algorithm and found that the immune score and the ESTIMATE score of the high-risk group were significantly higher than those of the low-risk group. These results indicated that ER stress could be associated with the involvement of the TME to regulate the development of KIRC. As reported, Treg cells could control NK cells, B cells, DC cells, and macrophages and inhibit tumor immune responses through regulating humoral and cell–cell contact (Tanaka and Sakaguchi, 2017). Another research study suggested that the enrichment of Treg cells could inhibit the anti-tumor immunoreactivity and was highly correlated with poor survival (Goschl et al., 2019). These findings were in line with our results of abundant Treg cells, macrophages, and B cells in the TME of KIRC patients with a high risk score. Finally, we identified the different expressions of immune checkpoints between the two risk groups to evaluate whether patients could benefit from ICI therapy. Therefore, the novel prognostic model probably could help assess the immune microenvironment of KIRC, and targeting ER stress might probably be a potentially valuable strategy for immunotherapy of KIRC patients.

However, there were still some limitations that warrant consideration. First, since the results of this study were based on bioinformatics and relied on public databases, there was no experimental validation of the results. Second, the performance of our prognostic model lacked validation in more independent databases. Finally, further study and complementary *in vivo* and *in vitro* experiments are necessary to confirm our findings.

## Conclusion

In conclusion, we constructed a novel prognostic model based on eight ER stress-related genes for KIRC patients and verified its independent prognostic value. We also clarified that this prognostic model was highly correlated with clinical characteristics and the immune microenvironment of KIRC patients. The novel prognostic model would help predict the prognosis of KIRC and provide a new orientation to further research studies on personalized immunotherapy in KIRC.

## Data Availability

The original contributions presented in the study are included in the article/Supplementary Material; further inquiries can be directed to the corresponding author.

## Abbreviations

DEGs, differentially expressed genes; ER, endoplasmic reticulum; ERAD, endoplasmic reticulum-associated degradation; GEO, Gene Expression Omnibus; GO, Gene Ontology; HR, hazard ratio; ICIs, immune checkpoint inhibitors; KIRC, kidney renal clear cell carcinoma; KEGG, Kyoto Encyclopedia of Genes and Genomes; LASSO, least absolute shrinkage and selection operator; MSigDB, Molecular Signatures Database; PPI, protein–protein interaction; RCC, renal cell carcinoma; ssGSEA, single-sample Gene Set Enrichment Analysis; TME, tumor microenvironment.
